# SynEL: A synthetic benchmark for entity linking

**DOI:** 10.1371/journal.pone.0339468

**Published:** 2026-01-08

**Authors:** Ilia Karpov, Alexander Kirillovich, Elisaveta Goncharova, Andrey Parinov, Alexander Chernyavskiy, Dmitry Ilvovsky, Natalia Semenova, Artyom Sosedka, Ekaterina Lisitsyna, Mikhail Belkin

**Affiliations:** 1 HSE University, Moscow, Russia; 2 Independent Researcher, Moscow, Russia; Shanghai Maritime University, CHINA

## Abstract

Large language models (LLMs) offer significant potential for constructing commonsense knowledge graphs from text, demonstrating adaptability across diverse domains. However, their effectiveness varies significantly with domain-specific language, highlighting a critical need for specialized benchmarks to assess and optimize knowledge graph construction sub-tasks like named entity recognition, relation extraction, and entity linking. Currently, domain-specific benchmarks are scarce. To address this gap, we introduce SynEL, a novel benchmark developed for evaluating text-based knowledge extraction methods, validated using customer support dialogues. We present a comprehensive methodology for benchmark construction, propose two distinct approaches for generating synthetic datasets, and evaluate accumulated hallucinations. Our experiments reveal that existing LLMs experience a significant performance drop, with micro-F1 scores decreasing by up to 25 absolute points when extracting low-resource entities compared to high-resource entities from sources like Wikipedia. Furthermore, by incorporating synthetic datasets into the training process, we achieved an improvement in micro-F1 scores of up to 10 absolute points. We publicly release our benchmark and generation code to demonstrate its utility for fine-tuning and evaluating LLMs.

## Introduction

Recent growth in the parameter count and training corpora size of large language models (LLMs) has greatly improved their ability to generate coherent text for specific tasks. A particularly promising application is the construction of commonsense knowledge graphs (KGs) from text, a task where LLMs show high adaptability across diverse domains. **Knowledge Graphs** (KGs) are structured representations of entities, their attributes, and semantic relationships serve as powerful resources for various NLP applications. Their popularity has surged due to advances in Graph-based Retrieval-Augmented Generation pipelines, which integrate graph-structured knowledge into the LLM’s context to improve understanding and reduce hallucinations during response text generation.

However, the effectiveness of LLMs in KG construction varies significantly depending on domain-specific language. This variability highlights the critical need for specialized benchmarks to assess and optimize key components of the process. Populating a knowledge graph from unstructured text typically involves several key steps [1–3]:

- **Named Entity Recognition** (NER): Identifying and classifying named entities (e.g., people, organizations) in text [4].
- **Relation Extraction** (RE): Identifying and classifying semantic relationships between recognized entities [5].
- **Coreference Resolution** (CR): Resolving different textual mentions that refer to the same real-world entity within a document or across multiple documents [1].
- **Entity Linking** (EL): Mapping recognized entities to their corresponding entries in an external knowledge base like Wikidata or DBpedia [6,7].
- **Knowledge Fusion**: Integrating and deduplicating extracted information to create a unified knowledge graph.
- **Quality Assessment & Validation**: Evaluating the accuracy of the extracted entities and relations and verifying integrity.

In this work, we focus on the first four steps, which LLMs can effectively address, and propose an approach for generating training datasets to fine-tune models for these tasks. Our approach is especially useful for handling **Low-Resource Entities** - entities underrepresented in the text corpora used for LLM training. In commonsense contexts, these often include small companies or non-public individuals, typically found in internal databases or public registers. Due to specific data formats, contemporary LLMs struggle to effectively process this information even when given access to such data sources.

We explore improving Entity Linking quality for **Closed Information Extraction** (cIE) tasks by using synthetic datasets for fine-tuning. We define cIE as NER→RE→EL process operating with (i) a closed list of relations (see [8]) and (ii) a closed list of entities. The output is a list of disambiguated triplets (subject, object, relation), which can be easily transformed into the Resource Description Framework (RDF) [9] format widely used in industry. Practical applications include automating customer base interactions and structuring internal company data.

Both relations and entities can be low-resource, but our preliminary experiments show that LLMs handle rare relations well after minimal fine-tuning. Therefore, we primarily evaluate model performance based on entity linking, which requires accurate NER and RE as prerequisites. Existing EL methods, trained on encyclopedic datasets like YAGO or Wikidata [10], focus on well-known public figures, which differs significantly from industrial use cases involving entities with similar names and contexts, such as in customer support dialogues.

We assert that EL models perform significantly worse on low-resource entities compared to high-resource entities. Fine-tuning on existing annotated data is often infeasible due to several shortcomings of existing datasets:

- Low frequency of low-resource entities in external datasets [11].
- Mismatch between relation types in external datasets and internal KGs.
- Discrepancy between the training dataset language and the target language for inference [12].
- Contradictory facts across different datasets.

These challenges can be overcome by generating a synthetic dataset that meets three key conditions:

- **Naturalness**: The dialogues must be as close to real conversations as possible.
- **Collection size**: The dataset must be large enough for both training and testing.
- **Markup**: Each dialogue must be annotated with mentioned entities and their standard identifiers.

The main **contributions** of our work are as follows:

- We propose a synthetic dataset generation method for training and validating low-resource entity linking models. Our method uses existing LLMs, incorporating known attributes, entities, and relations from knowledge graphs to create realistic texts and accurate annotations from existing knowledge graphs.
- We create a synthetically generated multilingual benchmark with various relation types suitable for cIE tasks in the financial sector [13,14]. We manually annotate a subsample of the generated benchmark to investigate model hallucinations and assess the error rate.
- We build a classical cIE evaluation pipeline demonstrating that fine-tuning on our synthetic data significantly improves quality for both GNN-based and LLM-based approaches.

This paper is structured as follows: we first review related work on synthetic dataset generation. Next, we describe our method for generating dialogues from knowledge graphs. We then validate the quality of the generated dialogues and analyze generation errors. Finally, we detail our experimental pipeline for assessing the dataset applicability for practical NLP tasks.

## Related work

Synthetic dataset generation typically involves three stages: (i) generation, (ii) curation, and (iii) evaluation [15].

**Generation** can be done via simple prompt engineering [16,17] or Multi-Step Generation, where a chain of simpler sub-tasks produces data step-by-step. The latter is more effective for complex reasoning and long contexts [18], and we adopt this two-step approach. Our method is similar to the strategy in [19], which introduced SynthIE and demonstrated the feasibility of using generated datasets for REBEL [20]. However, SynEL makes a distinct contribution by focusing on low-resource entities from non-encyclopedic domains, such as corporate registries. Unlike SynthIE, which leverages the rich context of Wikipedia, our benchmark simulates the more challenging industrial scenario of linking entities with sparse mentions. REBEL focuses on general relation extraction, whereas SynEL is tailored for the full cIE pipeline. Our work further differs by employing multiple generation strategies, using a classifier to filter artificial texts, and specifically targeting low-resource entities, whereas SynthIE mainly evaluates well-known entities richly represented in Wikidata.

**Curation**: Generated datasets often contain noise or harmful samples due to hallucinations or ambiguous prompts. Crowdsourcing is widely used for correction [21]. We control for hallucinations with human intervention, similar to [22] and [23]. We also tested a classifier for artificial text detection [24], but found that modern LLMs can generate high-quality text without extra filtering.

**Evaluation**: The traditional EL pipeline first identifies named entities and then links them [25]. Early methods used CNNs [26] or LSTMs [27,28] for context-mention encoding. More recent works like LlmLink propose dual-LLM frameworks for dynamic linking in long narratives [29]. However, low-resource entities often share similar contexts, making context-mention encoding less suitable. We instead perform Relation Extraction before Entity Linking, allowing us to use extracted facts for more accurate linking, including with graph models.

Evaluation models trained on synthetic data may overfit to generation artifacts. A potential solution is Domain Adversarial Neural Networks (DANN) [30] to train more robust models by aligning representations from different domains. Recent studies show this can improve F1-scores for classifying synthetic texts by ∼1.5% [31].

Entity linking models integrating structured data are described in [25]. Some use self-supervision, such as SS-AGA [32] and SelfKG [33]. Others focus on improving reasoning over knowledge graphs for downstream tasks like question answering [34]. We employ a self-supervised method based on SelfKG to produce robust entity representations. The availability of high-quality, domain-specific datasets, such as in finance [35], remains a key accelerator for such research.

## Synthetic dialogues generation method

In this section, we describe our methods for generating synthetic, annotated dialogues using LLMs. Each dialogue is a multi-turn conversation between a bank client and a customer-support agent, accompanied by in-text annotations of named-entity mentions. Every annotation specifies the entity type and, whenever applicable, the corresponding entity identifier in the target knowledge graph.

### Data source specification

DBpedia is an encyclopedic KG based on Wikipedia, containing descriptions of 4.8 million entities. Data derived from DBpedia can be used under the CC-BY-SA 3.0 license. The Public Company Register (EGRUL) is an enterprise KG with meta-information about Russian companies, maintained by the Federal Tax Service. It includes over 30 fields for 1,878,507 organizations. The DBpedia dataset is publicly available under a CC-BY-SA 3.0 license. Data from the Public Company Register (EGRUL) is subject to mandatory public disclosure under Russian Federal Law No. 129 and was handled in compliance with Federal Law No. 152 on personal data protection. For the pseudonymization-based generation method, all original data was anonymized by our industrial partner, and all entities inserted during the pseudonymization process are entirely fictional, bearing no resemblance to the original data, thus ensuring the privacy of all involved parties. All participants in the original dialogues provided consent for data analysis and sharing as part of their service agreement.

We evaluated only short organization names, excluding legal forms. Companies were labeled based on region, occupation, and financial results, with each having five nearest competitors. Key data source characteristics are in Table 1.

**Table 1:** Data source statistics.

Dataset	Language	Avg. Links	Median Polysemy
DBpedia	English	12	3±32
Public Company Register (EGRUL)	Russian	16	2±143

Characteristics in Table 1 were computed for 3,458 randomly selected companies from DBpedia and 3,794 from the Public Company Register (EGRUL). Median Polysemy shows there are 2 candidates per name in the Public Company Register (EGRUL) vs. 3 in DBpedia, but with vastly different standard deviations. Avg. Links is the average number of relations per entity. We filtered for frequent relationship types, adding a “is_competitor_of” relation for the Public Company Register (EGRUL) KG. The full list is in Table 1 in the S4 Appendix.

We conducted preliminary experiments with Mistral, SAIGA, GigaChat, and OpenAI GPT versions available in December 2023. We selected ‘gpt-3.5-turbo-1106’ as the generation model for both datasets, as its performance was comparable to GPT-4.0 at a lower cost.

### KG-based dialogues generation scheme

In this section, we describe our method for generating synthetic annotated dialogues using LLMs grounded in DBpedia and EGRUL knowledge graphs.

We initially considered a naïve approach in which an LLM first produces a dialogue and then identifies and annotates mentions of knowledge-graph entities. However, this approach suffers from two critical drawbacks. First, using such LLM-annotated dialogues as an evaluation set for entity linking would result in circular evaluation, since the same family of models would both generate the labels and be assessed on them. Second, the entities introduced by the LLM would often be fictional, making it impossible to ground them in a knowledge base for downstream EL tasks.

**Fig 1:**
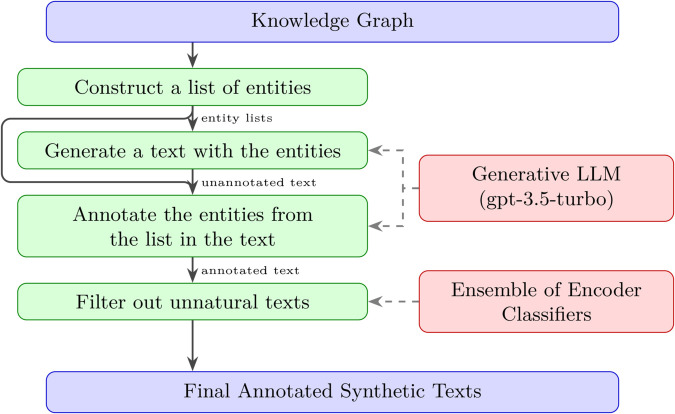
Key stages (green) of the knowledge graph (blue) based dialogue generation method.

To avoid these issues, we adopt an inverse approach (Fig 1). Instead of generating a dialogue and then discovering the entities within it, we first construct the answer to the EL problem—namely, a predefined set of entities that must appear in the dialogue—and only then ask the LLM to generate text that conforms to this predefined solution. More concretely, the method proceeds as follows: (i) we construct a list of knowledge-graph entities to be mentioned in the dialogue, each described by its name, type, and KG identifier; (ii) we prompt the LLM to compose a dialogue in which some entities from this list appear in natural contexts; and (iii) we further prompt the LLM to locate mentions of these entities in its own generated text and insert the corresponding annotations. As a result, the annotation task reduces to matching surface mentions of already known entities and attaching already known attributes (type and identifier), eliminating ambiguity and ensuring full grounding in the target knowledge graph.

#### DBpedia-based dialogues generation.

The prompt includes the company’s name (‘rdfs:label’), industry (‘dbo:industry’), location (‘dbo:headquarter’), and number of employees (‘dbo:numberOfEmployees’). The dialogue is required to mention several other related companies, sourced from ‘dbo:wikiPageWikiLink’. An example dialogue is in S1 Appendix (‘DBpedia-based dialogue’); the full prompt is in S2 Appendix (‘DBpedia-based dialogue’).

#### Public company register (EGRUL) dialogues generation.

Generating dialogues based on the Public Company Register (EGRUL) database follows a similar process. The prompt includes instructions to create a dialogue between a bank support service and a company representative, providing the company’s name, industry, location, and capital size. An example dialogue is in S1 Appendix (‘Company register-based dialogue’); the full prompt is in S2 Appendix (‘Company register-based dialogue’).

### Pseudonymization-based dialogues generation scheme

**Fig 2:**
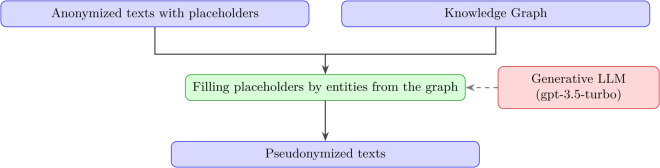
Filling placeholders (green) of the anonymized texts (blue) using a knowledge graph (blue).

This method uses transcripts of real conversations provided by an industrial partner. To ensure confidentiality, the transcripts were anonymized by replacing real entity mentions with placeholders (# and *). The process is as follows: we use ChatGPT to pseudonymize the dialogues by replacing placeholders with names of fictional entities. All generated entity names are fictional and do not correspond to the original anonymized data, ensuring full compliance with data privacy standards. Then, by comparing the anonymized and pseudonymized versions, we extract a list of the inserted entities, which serves as the dialogue’s annotation (Fig 2).

### Curation scheme

Generative models can introduce artifacts that negatively impact training. We use a model for detecting artificial texts based on an ensemble classifier approach [24], which achieved top results at the RuATD 2022 competition [36]. Our approach uses an ensemble of five independent models trained with 5-fold cross-validation. Predictions from each model on its hold-out set are averaged, and logistic regression model is trained on these scores to form the final ensemble. The models include:

- BERT-based models: ‘sberbank-ai/sbertlargenluru’, ‘sberbank-ai/ruBert-large’, ‘DeepPavlov/rubertbase-cased’ [37].
- mBART fine-tuned for summarization: ‘IlyaGusev/mbartrusumgazeta’ [38].
- Multilingual classification models: ‘MoritzLaurer/mDeBERTa-v3-base-mnli-xnli’ [39] and ‘DeepPavlov/xlm-roberta-large-en-ru-mnli’ [40].

The ensemble was trained on a genre-diverse corpus including social media, news, Wikipedia, the Russian National Corpus, and government reports. We observed that classification errors increase for short texts (< 18 tokens). To improve accuracy, we segment texts into fragments of full sentences (150-255 tokens). The overall artificiality score for a text is the maximum score across its fragments. We exclude the 20% of texts with the lowest confidence scores from further use.

### Generated dataset validation

#### Dataset evaluation.

To assess the quality of the generated EL annotations, we conducted a manual validation. Three undergraduate Computer Science students, supervised by a senior annotator (Ph.D. in CS), reviewed a random sample of 800 dialogues. They corrected annotations by inserting missing entity links and flagging incorrect ones. An annotation was deemed incorrect if (1) the text span was not a named entity, (2) the entity type was wrong, or (3) the entity link was incorrect. The senior annotator reviewed all corrections. The final validated set formed a gold standard against which the original annotations were evaluated using precision, recall, and F1 score. Results for DBpedia-based and Public Company Register (EGRUL)-based dialogues are in Tables 2 and 3. All manual annotations are available on GitHub.

**Table 2:** Validation of DBpedia-based dialogues *(400 dialogs, 128,647 words sample).*

Entity type	TP	FP	FN	R	P	F1
Companies	2070	13	17	0.992	0.994	0.993
Industries	1113	45	117	0.905	0.961	0.932
Locations	48	0	9	0.842	1	0.914
All	3231	58	143	0.958	0.982	0.970

**Table 3:** Validation of public company register (EGRUL)-based dialogues *(400 dialogs, 88,932 words sample).*

Entity type	TP	FP	FN	R	P	F1
Companies	1559	10	1	0.999	0.994	0.996
Industries	239	52	54	0.816	0.821	0.818
Locations	403	12	15	0.964	0.971	0.968
All	2201	74	70	0.969	0.967	0.968

#### Inter-annotator agreement.

To evaluate the consistency of manual validation, we measured inter-annotator agreement (IAA). We used the pairwise F1 measure, a common metric for NER and EL evaluation [41,42], as Cohen’s kappa is less suitable for sequence-based tasks. A set of 200 randomly selected dialogues was assigned to all three annotators for independent review. Agreement was assessed by calculating the pairwise F1 score for each pair of annotators, treating one’s corrections as the gold standard and evaluating the other’s against it. An annotation was considered correct if it had the same entity type, link, and an overlapping span. The final IAA score is the average of the three pairwise F1 scores. IAA results for DBpedia-based and Public Company Register (EGRUL)-based dialogues are in Tables 4 and 5.

**Table 4:** Inter-annotator agreement for validation of DBpedia-based dialogues *(100 dialogues sample).*

Entity type	TP	FP	FN	R	P	F1
*Annotator#1 vs annotator#2*						
Companies	524	3	0	0.994	1.000	0.997
Industries	276	29	8	0.905	0.972	0.937
Locations	17	0	0	1.000	1.000	1.000
All	817	32	8	0.962	0,990	0,976
*Annotator#1 vs annotator#3*						
Companies	522	5	2	0.991	0.996	0.993
Industries	264	20	20	0.930	0.930	0.930
Locations	308	10	12	0.963	0.969	0.966
All	802	26	23	0.969	0.969	0.970
*Annotator#2 vs annotator#3*						
Companies	526	1	1	0.998	0.998	0.998
Industries	272	12	32	0.958	0.895	0.925
Locations	16	1	1	0.941	0.941	0.941
All	814	14	14	0.983	0.960	0,971
*Average*						
Companies				0.994	0.998	0.996
Industries				0.931	0.832	0.931
Locations				0.961	0.961	0.961
All				0.971	0.974	0.973

**Table 5:** Inter-annotator agreement for validation of public company register (EGRUL)-based dialogues *(100 dialogues sample).*

Entity type	TP	FP	FN	R	P	F1
*Annotator#1 vs annotator#2*						
Companies	399	1	0	0.998	1.000	0.999
Industries	60	10	11	0.857	0.845	0.851
Locations	109	2	5	0.982	0.956	0.969
All	568	13	16	0.978	0,973	0,975
*Annotator#1 vs annotator#3*						
Companies	399	1	0	0.998	1.000	0.999
Industries	62	14	10	0.816	0.861	0.838
Locations	112	4	2	0.966	0.982	0.974
All	573	19	12	0.968	0.979	0.974
*Annotator#2 vs annotator#3*						
Companies	400	0	0	1.000	1.000	1.000
Industries	66	10	4	0.868	0.943	0.904
Locations	108	8	3	0.931	0.973	0.952
All	574	18	7	0.970	0.988	0,979
*Average*						
Companies				0.998	1.000	0.999
Industries				0.847	0.883	0.864
Locations				0.960	0.971	0.965
All				0.972	0.980	0.976

#### Error analysis.

Our validation revealed the following error causes:

- **Errors in the Knowledge Graph (3.17%):** For example, DBpedia misclassified ‘dbpedia:Holding’ as an industry.
- **Incorrect Span Detection (17.46%):** The LLM annotated a text span that was not a named entity.
- **Incorrect Entity Type Assignment (20.63%):** The LLM assigned the wrong type to a correct entity span.
- **Incorrect Linking (3.17%):** The LLM linked a correct span to the wrong entity in the KG.
- **Hallucinated Entity (6.35%):** The LLM generated a mention of an entity not present in the KG and not requested in the prompt.
- **Missed Annotation (49.21%):** The LLM failed to identify a known named entity it was prompted to annotate.

These statistics show that direct entity linking errors are rare due to our reverse generation approach. The most common issue is the model failing to include a requested entity in the dialogue.

## Experiment design

This section describes our use of the dataset for two purposes: (i) as a benchmark to evaluate existing EL methods, and (ii) as training data to fine-tune some of these methods. The English DBpedia-based dataset represents a high-resource scenario, while the Russian dataset from the tax service registry serves as a low-resource example.

Our model selection includes a diverse range of architectures and sizes to provide a comprehensive evaluation. We include a state-of-the-art proprietary model (GPT-4o) as a high-performance baseline, alongside popular open-source models of varying sizes (Mistral-24B, Falcon-11B) to assess performance across different resource constraints. The inclusion of encoder-based models (BERT, XLM-RoBERTa) and GNNs allows for a comparison between LLM-native pipelines and more traditional, fine-tunable approaches.

We split the knowledge graphs and corresponding dialogues into 80/20 train/test sets. The test set is used to evaluate:

- End-to-end models like DBpedia Spotlight (dbpedia-spotlight.org) and GPT-4o.
- Zero-shot decoder- and encoder-based models for NER and EL.
- Fine-tuned encoder-based models and Graph Neural Network (GNN) models.

**Fig 3:**
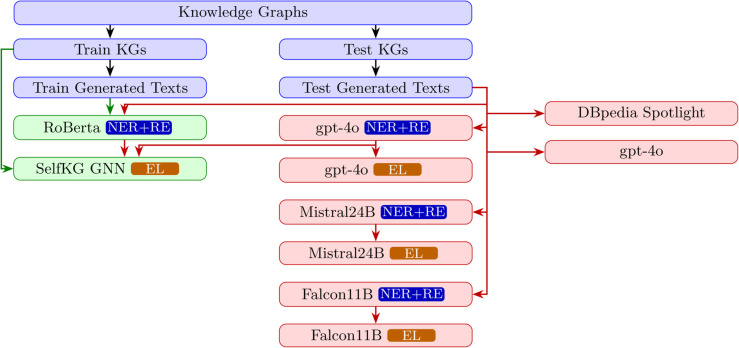
Usage of generated texts for fine-tuning (green) and evaluating (red) various models.

Approaches not using our synthetic data for fine-tuning are highlighted in red in Fig 3, while those that do use are in green.

### Named entity recognition and relation extraction

We employ three NER approaches: (1) explicit queries to an LLM, (2) zero-shot entity extraction with a pre-trained encoder, and (3) fine-tuning an encoder on our generated dataset.

**NER & RE using Decoder based Language Models**

We use separate NER (step 1) and RE (step 2) prompts with decoder models, including gpt-4o (‘gpt-4o-2024-08-06’), Mistral-24B (Mistral-Small-24B-Instruct-2501), and Falcon-11B (falcon-11B) [43]. For all LLM experiments, to ensure reproducibility, we set temperature to 0.7, top_p to 0.9, and the maximum output length to 2048 tokens, unless otherwise specified. For Falcon-11B, that often ignored formatting instructions, up to 10 generation attempts were made per prompt to obtain a response in the correct format. System prompts are provided in S4 Appendix for reproducibility.

**NER & RE using Encoder based Language Models**

This standard approach for token classification involves creating pairs of entities after NER and classifying the relationship between them. We frame this as a multiclass classification problem, including a “no relation” category. To improve upon simple text classification, we mark entity spans with special tokens (*E*1_*start*_, *E*1_*end*_, etc.) and concatenate the embeddings of the start tokens (*E*1_*start*_, *E*2_*start*_) as input to a classification head, which has shown superior performance [44]. We also use two modifications:

- **Input Preprocessing:** We add entity type tokens to the input (e.g., “E1 ORG bank /E1”) to provide additional cues.
- **False Positive Mitigation:** We use a preprocessing step based on entity type constraints to filter invalid relationship predictions [45].

To fine-tune encoders, we used several public datasets. For English, we used **REFinD** [46], a financial dataset with 20 relation types, and **FinRED** [47], a larger dataset from news articles with 29 relations from Wikidata. For Russian, the closest dataset is **NEREL** [48], compiled from Russian WikiNews, which features nested named entities.

Our key hypothesis is that public datasets do not sufficiently cover low-resource entities. We augment these datasets with our generated dialogues and fine-tune an encoder model on the combined data.

Table 6 shows the results of fine-tuning encoders for the NER task. For English, we used a BERT-base model trained on a subset of Clean-CoNLL [49] plus 1,000 of our synthetic dialogues. For Russian, we used an XLM-RoBERTa-base model trained on our synthetic dialogues.

**Table 6:** Results of encoder models fine-tuning for NER task.

NER	R	P	F
*DBpedia*			
BERT-base (CoNLL)	0.46	0.63	0.51
BERT-base (CoNLL + *1,000 synth. texts*)	0.48	0.67	0.53
*Russian*			
XLM-Roberta-base (*1,000 synth. texts*)	0.56	0.68	0.61
XLM-Roberta-base (*5,000 synth. texts*)	0.75	0.81	0.78
XLM-Roberta-base (*10,000 synth. texts*)	0.79	0.84	0.81

**Dataset Statistics:** The DBpedia dataset consists of 1,000 texts (5,569 samples of 512 tokens). The CoNLL dataset has 47,959 samples. The Russian dataset has 10,000 dialogues (32,700 samples). **Training Details:** Models were trained for 5 epochs using the AdamW optimizer with a learning rate of 5e-3 and a batch size of 4. The fine-tuning method uses positional embeddings and special entity-marking tokens, following [50,51].

### Entity linking

At the EL step, we link entities and their extracted relationships to records in a knowledge base. All entities detected in the text are supposed to exist in the KG. We propose two approaches: one using an LLM and one using a Graph Neural Network (GNN).

**Entity Linking using Decoder based Language Model**

We prompt gpt-4o to select the best entity from a list of candidates, given facts extracted from the text, based on the method from [52]. We test two strategies:

1. **Candidate List:** The model chooses the best match from a list of up to 100 candidates, each described by its context.
2. **Pairwise:** The model determines if the entity from the text and a single candidate from the KG are the same. The process stops at the first positive match.

A comparison is presented in Sect Entity linking with LLMs: Candidate list vs pairwise for decoder LLMs.

**Entity Linking using Graph Neural Network.**

**Fig 4:**
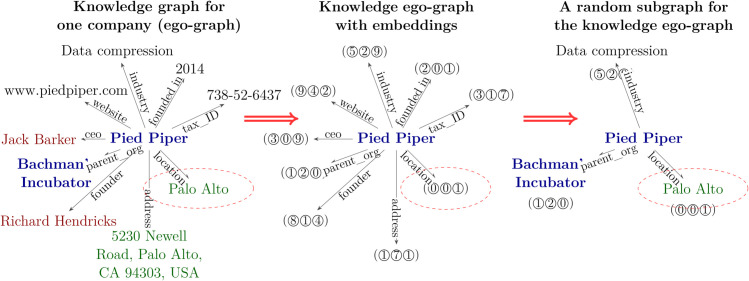
The training scheme for the vector representation of the ego-graph. Positive samples are constructed by contrasting sparse (15% nodes) and rich (85% nodes) subgraphs from the original ego-graph to simulate text extraction vs. KG data.

Here, we train a self-supervised graph encoder directly on the knowledge graphs, not the generated texts, using an unsupervised approach from [33]. As shown in Fig 4, the model learns to determine if two ego-graphs belong to the same entity. Positive samples are created by taking two different subgraphs of the same entity’s ego-graph (one large, 85% of nodes; one small, 15% of nodes) to simulate a rich KG record versus sparse information from text. Negative samples are drawn from random entities with the same name. Node names are encoded using the LaBSE model [53]. The model is trained with a contrastive loss function, similar to methods like MoCo [54] and SimCLR [55].

## Evaluation

We use two data sources: DBpedia (high-resource, English) and the Public Company Register (EGRUL) (low-resource, Russian). All experiments evaluate final Entity Linking quality using the F1 score.

We perform pairwise t-tests on the full test sets to assess the statistical significance of performance differences between models. We obtained p-values < 0.05 for all pairwise comparisons, confirming that the observed differences are statistically significant, except for the comparison between Mistral24B and gpt-4o on DBpedia.

### Entity linking with LLMs: Candidate list vs pairwise for decoder LLMs

This experiment compares EL quality when providing a list of candidates versus pairwise comparison in the prompt. We evaluate EL only for the central entity of each dialogue. Linking all entities mentioned dramatically reduces the speed of LLM-based approaches.

The results in Table 7 show that pairwise prompting yields better results, suggesting that even powerful models like gpt-4o struggle to handle long factual contexts with many candidates. However, the pairwise approach is computationally expensive, making GNNs an attractive alternative.

**Table 7:** Comparison of multiple/pairwise object prompting for EL with LLMs for public company register (EGRUL) knowledge graph.

EL (Central Entity)	NER + RE	R	P	F
gpt-4o (Candidate List)	XLM-RoBERTa	0.488	0.603	0.54
gpt-4o (Candidate List)	Finetuned XLM-RoBERTa	0.558	0.667	0.608
gpt-4o (Candidate List)	gpt-4o	0.541	0.672	0.6
gpt-4o (Pairwise)	XLM-RoBERTa	0.421	0.421	0.421
gpt-4o (Pairwise)	Finetuned XLM-RoBERTa	0.671	0.671	**0.671**
gpt-4o (Pairwise)	gpt-4o	0.351	0.351	0.351

### Entity linking with GNNs: Central entity vs all entities

We compare EL performance for linking only the **central entity** versus linking **all entities** in the text for the Public Company Register (EGRUL) dataset. The central entity has a rich context, while other entities are often mentioned only by name.

Surprisingly, linking all entities yielded higher F1 scores (Table 8). This can be explained because successfully linking the central entity often leads to successful linking of other mentioned entities, boosting the overall metrics.

**Table 8:** Comparison of different GNN setup for synthetic dataset made from public company register (EGRUL) knowledge graph.

EL	NER + RE	R	P	F
*All Entities*				
GNN	Roberta	0.521	0.669	0.586
GNN	Finetuned XLM-RoBERTa	0.682	0.667	**0.674**
GNN	gpt-4o	0.616	0.538	0.575
*Central Entity*				
GNN	Roberta	0.535	0.389	0.45
GNN	Finetuned XLM-RoBERTa	0.711	0.6	0.651
GNN	gpt-4o	0.537	0.319	0.4

### Unsupervised LLM for entity linking comparison

In this experiment, we compare several LLMs of different sizes on both datasets. We use DBpedia Spotlight and a single-prompt gpt-4o as baselines.

As shown in Table 9, all unsupervised language models perform well on the DBpedia dataset, as they were trained on Wikipedia. DBpedia Spotlight acts as a strong baseline with an F1 score of 0.81, while the end-to-end gpt-4o model improves this to 0.83. The poor results for Falcon11B are primarily due to its failure to generate a valid response even after 10 requests, highlighting the importance of using instruction-tuned models for pipeline tasks. In contrast, the performance of these models on the Public Company Register (EGRUL) data is dramatically worse. The best zero-shot result from gpt-4o is a low 0.351 F1 score. Most errors from Mistral24B and Falcon11B are related to the entity recognition step; for example, Falcon11B often misses simple entities or transliterates them to English despite instructions. An interesting finding is Mistral24B’s low rate of false positives: it rarely misidentifies an entity but often expresses uncertainty when context is insufficient.

**Table 9:** Comparison of unsupervised LLM for entity linking.

EL	NER + RE	R	P	F
*DBpedia*				
Mistral24B	Mistral24B	0.81	0.99	0.89
Falcon11B	Falcon11B	0.78	0.83	0.80
gpt-4o	gpt-4o	0.82	0.97	0.89
single prompt gpt-4o		0.80	0.87	0.83
DBpedia Spotlight		0.77	0.86	0.81
*Public Company Register (EGRUL)*				
Mistral24B	Mistral24B	0.099	0.99	0.18
Falcon11B	Falcon11B	0.017	0.25	0.11
gpt-4o	gpt-4o	0.351	0.351	0.351
single prompt gpt-4o		0.126	0.376	0.189

### Finetuned encoder models vs unsupervised decoder LLMs on synthetic data

Here, we compare the best models on both the high-resource (DBpedia) and low-resource (Public Company Register (EGRUL)) datasets.

**DBpedia:** Table 10 shows that while all methods perform well on this high-resource dataset, fine-tuning provides a clear advantage. Using a GNN for EL with a standard BERT encoder for NER+RE yields an F1 score of 0.86. Fine-tuning the encoder on our generated texts increases performance to 0.89, the highest result on the DBpedia dataset.

**Table 10:** Results of the experiments on synthetic data.

EL	NER + RE	R	P	F
*DBpedia*				
GNN	BERT	0.78	0.95	0.86
GNN	Finetuned BERT	0.83	0.95	**0.89**
GNN	gpt-4o	0.85	0.77	0.83
*Public Company Register (EGRUL)*				
GNN	XLM-RoBERTa	0.535	0.389	0.45
GNN	Finetuned XLM-RoBERTa	0.711	0.6	0.651
GNN	gpt-4o	0.537	0.319	0.4
gpt-4o	XLM-RoBERTa	0.421	0.421	0.421
gpt-4o	Finetuned XLM-RoBERTa	0.671	0.671	**0.671**
gpt-4o	gpt-4o	0.351	0.351	0.351
single prompt gpt-4o		0.126	0.376	0.189

**Public Company Register (EGRUL):** We use a single-prompt gpt-4o model as a baseline. The model is asked to extract organizational facts and find their tax numbers. Despite having internet search capabilities, this approach achieves an F1 score of only 0.189, as it fails to filter search results and match records to the known facts. Simply using graph information with a standard encoder improves the F1 score to 0.45. The most significant improvement comes from fine-tuning the encoder on the specifics of our synthetic dataset, which boosts the F1 score to 0.671.

### Finetuned encoder models vs unsupervised decoder LLMs on real data

To evaluate performance on real data, we used a dataset of 332 anonymized customer support dialogues in Russian. We automatically filled anonymized placeholders with relevant company data from our Public Company Register (EGRUL) dataset and manually filtered for logical consistency. These dialogues were not used for training. Table 11 shows the EL quality of the best models on this real-world dataset. Analysis revealed that many dialogues lacked rich disambiguation context, limiting the pipeline’s potential. However, the method fine-tuned on synthetic data still outperformed the zero-shot gpt-4o, illustrating the practical value of our approach. The best performance (F1 0.533) was achieved by combining gpt-4o for EL with a fine-tuned XLM-RoBERTa for NER+RE, mainly due to improved NER+RE performance. We found that most EL errors occurred in dialogues containing only the company name, where gpt-4o was often uncertain. The fine-tuned encoder was more effective at identifying tax numbers and relationships, especially when fragmented across tokens, leading to better performance.

**Table 11:** Results of the experiments on real data.

EL	NER + RE	R	P	F
gpt-4o	gpt-4o	0.355	0.76	0.484
gpt-4o	Finetuned XLM-RoBERTa	0.405	0.782	**0.533**

## Discussion

Our experiments highlight the limitations of using generalized models like gpt-4o for low-resource datasets without fine-tuning. The performance gap between encyclopedic (DBpedia) and low-resource (Public Company Register (EGRUL)) KGs exceeds 40 F1 points on synthetic data. We believe real-data performance could be further improved if the synthetic generation process more closely matched the distribution of entities and relationships in real-world dialogues. On our synthetic data, different EL models (GNN vs. LLM) produced relatively similar results, though GNNs struggled with texts that contained explicit identifiers or only a company name. All models performed significantly worse on the low-resource Public Company Register (EGRUL) dataset, underscoring the challenges of linking entities that are not well-represented in an LLM’s pre-training data. Fine-tuning is critical for improving baseline results in low-resource settings. The models tested on the Public Company Register (EGRUL) dataset showed significant gains from fine-tuning. Even on real data, where the context was often sparse, fine-tuning provided a 5-percentage-point improvement. In conclusion, these results emphasize that fine-tuning remains essential for enhancing entity linking quality, particularly in low-resource scenarios. This study illustrates the limitations of relying solely on generalized models for specialized domains and highlights the importance of domain-specific training to bridge the performance gap.

## Limitations

Despite the promising results, our study has several limitations.

**Knowledge Graph Quality:** Our generation process relies on an existing knowledge graph. We did not study the impact of errors or contradictions within the source KG on the quality of the generated benchmark and downstream model performance. The accuracy of the source KG is a critical prerequisite.

**Domain Generalizability:** While the proposed method is designed to be domain-agnostic, we have only validated it on customer support dialogues in the financial sector. Further research is needed to confirm its effectiveness in other domains (e.g., medical, legal) which may have different linguistic patterns and entity complexities.

**Potential for Bias:** The synthetic data generated by an LLM may inherit biases present in the LLM’s own training data. This could lead to a lack of diversity in the generated dialogues or the reinforcement of stereotypes, which might affect the robustness of models trained on this data.

**Real-World Generalization:** While we evaluated our models on a small set of real dialogues, a more extensive evaluation is needed to fully assess the generalization gap between performance on synthetic data and real-world, in-the-wild scenarios. The distribution of entities and relations in our synthetic dataset might not perfectly match that of real conversations.

**Closed-Set Evaluation:** Our evaluation is confined to a closed set of entities and relations defined by the source KG. The performance on open-set information extraction, where new or out-of-KG entities appear, remains an open question.

## Conclusion

In this paper, we propose a methodology for creating a benchmark dataset to train and validate an entity linking pipeline. Our approach uses LLMs to synthetically generate naturalistic dialogues, providing sufficient and high-quality data for training NER and EL modules. We found this approach valuable for evaluating existing pipelines and retraining them for specific domains, such as the financial sector. Our study utilized two generation approaches: one based on knowledge graphs (DBpedia, Public Company Register (EGRUL)) and the other on pseudonymizing real conversations. Both yielded high-quality annotations and natural dialogues. Our results indicate that synthetic data is a promising solution for NLP tasks, particularly in data-scarce scenarios. Future work will focus on:

1. expanding our method by integrating a variety of data sources and exploring its application across different domains;
2. generating cross-lingual information extraction benchmarks using existing knowledge graphs;
3. incorporating synthetic data from different LLMs using domain adaptation methods.

The dataset was published on Zenodo (https://doi.org/10.5281/zenodo.11470053) and the code is available on GitHub (https://github.com/alik-kirillovich/synel).

## Supporting information

S1 Appendix Examples of annotated dialogues automatically generated from the DBpedia and Companies Register knowledge graphs. (PDF)

S2 Appendix Examples of prompts used to generate annotated dialogues from the DBpedia and Public Company Register knowledge graphs. (PDF)

S3 Appendix Method for extracting entities from pseudonymization-based dialogues. (PDF)

S4 Appendix Named entity recognition and relation extraction parameters. (PDF)
