# Supplementary material for: SynEL: A synthetic benchmark for entity linking
Source: PLoS One. 2026 Jan 8;21(1):e0339468. doi: 10.1371/journal.pone.0339468 (PMC12782364; doi:10.1371/journal.pone.0339468)
Supplement: S1 Appendix — (PDF) [file pone.0339468.s001.pdf]

S1 Appendix: Generated Dialogue Examples

This appendix contains examples of annotated dialogues automatically generated from the DBpedia and Companies Register knowledge graphs.

DBpedia-based dialogue example

```
Dialogue #191 "Cameco":

Companies:
c0. Cameco
c4. Centerra Gold
c5. Westinghouse Electric Company

Industries:
i0. Mining
i4. Nuclear fuel

Locations:
l0. Saskatchewan

--Dialogue:--

Bank Customer Support: Thank you for calling the bank customer support.
How can I assist you today?

<c0>Cameco</c0> Representative: Hi, I'm calling from
<l0>Saskatchewan</l0> and I need to set up an ACH payment for our
company.

Bank Customer Support: Sure, I can help you with that. Can you please
provide me with your company's information and the details of the ACH
payment you'd like to set up?

<c0>Cameco</c0> Representative: Yes, our company, <c0>Cameco</c0>, is a
major player in the <i0>Mining</i0> industry, and we have 3300
employees. We need to set up recurring ACH payments for our suppliers
and contractors.

Bank Customer Support: Understood. Before we proceed, I just need to
confirm some security details. Can you please verify your account
information?

<c0>Cameco</c0> Representative: Of course. Our account number is
[account number].

Bank Customer Support: Thank you. Now, in terms of the ACH payments, do
you have specific suppliers and contractors in mind that you'll be
making payments to?

<c0>Cameco</c0> Representative: Yes, we work with companies like
<c4>Centerra Gold</c4> and <c5>Westinghouse Electric Company</c5> in
```

```
the <i0>Mining</i0> and <i4>Nuclear fuel</i4> industries.

Bank Customer Support: Got it. We'll need the banking information for
each of these suppliers and contractors to set up the ACH payments.
Also, do you have a preferred frequency for these payments?

<c0>Cameco</c0> Representative: We typically make payments to our
suppliers and contractors on a monthly basis, so setting up a monthly
recurring ACH payment would be ideal.

Bank Customer Support: Understood. I'll need to gather the necessary
details from you to set up the ACH payments. In the meantime, is there
anything else I can assist you with?

<c0>Cameco</c0> Representative: No, that's all for now. Thank you for
your help.

Bank Customer Support: You're welcome. I'll begin the process of
setting up the ACH payments and will be in touch if I need any further
information. Thank you for choosing our bank for your financial needs.

<c0>Cameco</c0> Representative: Thank you. Goodbye.
```

Companies register-based dialogue example

```
Dialog #5 «ООО "СЗ ККПД-ИНВЕСТ"»:

Companies:
c0. ООО "СЗ ККПД-ИНВЕСТ"
c1. ООО "ИВАСТРОЙ"
c2. ООО "ДОРСТРОЙСИСТЕМ"

Industries:
i0. Покупка и продажа собственного недвижимого имущества

Locations:
l2. Ростовская область

--Dialog:--

[Customer]: Добрый день! Я представитель <c0>ООО "СЗ ККПД-ИНВЕСТ"</c0>
и хочу обсудить с вами вопрос оплаты налогов.

[Support]: Добрый день! Конечно, мы готовы помочь. Какие именно налоги
вас интересуют?

[Customer]: Мы хотели бы уточнить процедуру оплаты налога на прибыль.
Мы занимаемся <i0>покупкой и продажей собственного недвижимого
имущества</i0>, и нам важно правильно оформить все налоговые
обязательства.
```

[Support]: Понятно. Для начала, вам необходимо учесть, что налог на прибыль в <l2>Ростовской области</l2> составляет 20%. Вы должны будете подать декларацию и оплатить налог до 28 марта следующего года.

[Customer]: Спасибо за информацию. Мы также хотели бы обсудить возможность открытия расчетного счета для удобства проведения финансовых операций.

[Support]: Конечно, мы можем помочь вам с этим. Для открытия расчетного счета вам необходимо предоставить определенный пакет документов. Мы можем отправить вам список необходимых документов и помочь с оформлением.

[Customer]: Отлично, будем ждать информацию от вас. Еще один вопрос: мы планируем провести крупные инвестиции в развитие нашего бизнеса. Какие условия кредитования предлагает ваш банк?

[Support]: Мы предлагаем различные программы кредитования для предприятий. Мы можем обсудить ваши потребности и подобрать оптимальное решение для вас.

[Customer]: Спасибо, это звучит интересно. Мы также сотрудничаем с компаниями <c1>ИВАСТРОЙ</c1> и <c2>ДОРСТРОЙСИСТЕМ</c2>, и нам важно, чтобы наши финансовые операции были максимально удобными для всех сторон.

[Support]: Мы готовы обсудить все детали сотрудничества с вашими партнерами и предложить им выгодные условия обслуживания.

[Customer]: Отлично, мы оцениваем ваше готовность к индивидуальному подходу. Спасибо за помощь, мы будем ждать информацию по налогам и открытию счета.

[Support]: Пожалуйста, обращайтесь, мы всегда готовы помочь.
