# Supplementary material for: SynEL: A synthetic benchmark for entity linking
Source: PLoS One. 2026 Jan 8;21(1):e0339468. doi: 10.1371/journal.pone.0339468 (PMC12782364; doi:10.1371/journal.pone.0339468)
Supplement: S2 Appendix — (PDF) [file pone.0339468.s002.pdf]

S2 Appendix: Dialogue Generation Prompts

This appendix contains examples of prompts used to generate annotated dialogues from the DBpedia and Public Company Register knowledge graphs. The values inserted into the prompt templates are highlighted in blue.

DBpedia-based dialogue

Here is an example prompt for generating an annotated dialogue based on the DBpedia knowledge graph:

Consider a company called ‘Cameco’, operating in the industry called ‘Mining’. It is based in Saskatchewan and has 3300 employees. Write a long dialogue between a company’s representative and a bank customer support. The main topic of the dialogue is arch payment setup. The dialogue should reflect the industry the company operates in, where the company is based, and the size of the company. The dialogue should also mention the company called ‘Uranium Participation Corporation’ operating in the industry called ‘Financial services’, the company called ‘Brookfield Renewable Partners’ operating in the industry called ‘Renewable power’, the company called ‘Bruce Power’ operating in the industry called ‘Electricity generation’, the company called ‘Centerra Gold’ operating in the industry called ‘Mining’, and the company called ‘Westinghouse Electric Company’ operating in the industry called ‘Nuclear fuel’. The dialogue must not mention any other companies, organizations and legal entities. The dialogue must not mention any industries other than the following: Mining, Financial services, Renewable power, Electricity generation, Nuclear fuel. The dialogue must not mention any locations other than the following: Saskatchewan. The company’s representative is nervous and hesitant. In the generated dialogue all the mentions of company called ‘Cameco’ must be enclosed by <c0> and </c0> tags (e.g. ‘I work in <c0>Cameco</c0>’), all the mentions of company called ‘Uranium Participation Corporation’ must be enclosed by <c1> and </c1> tags, all the mentions of company called ‘Brookfield Renewable Partners’ must be enclosed by <c2> and </c2> tags, all the mentions of company called ‘Bruce Power’ must be enclosed by <c3> and </c3> tags, all the mentions of company called ‘Centerra Gold’ must be enclosed by <c4> and </c4> tags, all the mentions of company called ‘Westinghouse Electric Company’ must be enclosed by <c5> and </c5> tags. In the generated dialogue all the mentions of the Mining industry must be enclosed by <i0> and </i0> tags, all the mentions of the Financial services industry must be enclosed by <i1> and </i1> tags, all the mentions of the Renewable power industry must be enclosed by <i2> and </i2> tags, all the mentions of the Electricity generation industry must be enclosed by <i3> and </i3> tags, all the mentions of the Nuclear fuel industry must be enclosed by <i4> and </i4> tags. In the generated dialogue all the mentions of the Saskatchewan location must be enclosed by <l0> and </l0> tags.

Public Company Register-based dialogue

Generating an unannotated dialogue

Here is an example prompt for generating an unannotated dialogue based on the Companies Register knowledge graph:

```
Рассмотрим компанию ООО "СЗ ККПД-ИНВЕСТ", сфера деятельности которой:
Покупка и продажа собственного недвижимого имущества. Компания
расположена по адресу Ростовская область, г. Ростов-На-Дону, ул.
Социалистическая, д. 74, офис 19А, 11 этаж и имеет капитал 2300000000
рублей. Напиши длинный диалог между представителем компании и службой
поддержкой банка. Основная тема диалога: оплата налогов, однако в
диалоге должны обсуждаться также и другие темы. Диалог должен отражать
сферу деятельности компании, ее местоположение и размер капитала.
Местоположение компании может упоминаться как в полном виде (с
регионом, городом и т.д.), так и в сокращенном (например, только улица
и дом). Также в диалоге должны упоминаться следующие компании: ИВАСТРОЙ
и ДОРСТРОЙСИСТЕМ. Представитель компании энергичный. В сгенерированном
диалоге реплика представителя компании начинается со слова
«[Customer]:» (например, «[Customer]: Да, конечно.»), а реплика службы
поддержки начинается со слова «[Support]: » (например, «[Support]: Да,
конечно.»).
```

Annotating the generated dialogue

Here is an example prompt for annotating the unannotated dialog generated by the previous prompt:

```
Consider the dialog in Russian below. Edit the dialogue as follows.
Find all the mentions of the company called ‘ООО "СЗ ККПД-ИНВЕСТ"’ and
enclose them between <c0> and </c0> tags (for example, ‘<c0>ООО "СЗ
ККПД-ИНВЕСТ"</c0>’). Find all the mentions of the company called
‘ИВАСТРОЙ’ and enclose them between <c1> and </c1> tags (for example,
‘<c1>ИВАСТРОЙ</c1>’). Find all the mentions of the company called
‘ДОРСТРОЙСИСТЕМ’ and enclose them between <c2> and </c2> tags (for
example, ‘<c2>ДОРСТРОЙСИСТЕМ</c2>’). Find all the mentions of the area
of activity (сфера деятельности) called ‘Покупка и продажа собственного
недвижимого имущества’ and enclose them between <i0> and </i0> tags.
(The field of activity does not have to be mentioned under its full
name). Find all the mentions of a place located at the address
‘Ростовская область, г. Ростов-На-Дону, ул. Социалистическая, д. 74,
офис 19А, 11 этаж’ and enclose them between <l0> and </l0> tags. (The
address can be in either full or abbreviated form). Find all the
mentions of the settlement called ‘г. Ростов-На-Дону’ which are not
part of the address ‘Ростовская область, г. Ростов-На-Дону, ул.
Социалистическая, д. 74, офис 19А, 11 этаж’ and enclose them between
<l1> and </l1> (e.g. ‘<l1>г. Ростов-На-Дону</l1>’). Find all the
mentions of the region called ‘Ростовская область’ which are not part
of the address ‘Ростовская область, г. Ростов-На-Дону, ул.
Социалистическая, д. 74, офис 19А, 11 этаж’ and enclose them between
<l2> and </l2> (e.g. ‘<l2>Ростовская область</l2>’). Find all the
mentions of the street address called ‘ул. Социалистическая, д. 74,
офис 19А, 11 этаж’ which are not part of the address ‘Ростовская
```

```
область, г. Ростов-На-Дону, ул. Социалистическая, д. 74, офис 19А, 11
этаж' and enclose them between <l0> and </l0> (e.g. '<l0>ул.
Социалистическая, д. 74, офис 19А, 11 этаж</l0>').

The dialog: {The text of the dialog generated by the previous prompt}
```
