# Supplementary material for: SynEL: A synthetic benchmark for entity linking
Source: PLoS One. 2026 Jan 8;21(1):e0339468. doi: 10.1371/journal.pone.0339468 (PMC12782364; doi:10.1371/journal.pone.0339468)
Supplement: S3 Appendix — (PDF) [file pone.0339468.s003.pdf]

S3 Appendix: Extracting entities from pseudonymization-based dialogues

In this appendix, we describe the approach, used to extract a list of entities that were inserted instead of the placeholder in pseudonymization-based dialogues.

Initially, we planned to extract the inserted entities based on an approach that involves analyzing the changes between the anonymized and pseudonymized text. It was assumed that all added text fragments found in this list of changes could be considered as names of the inserted entities. However, experiments showed that this assumption was incorrect. During the pseudonymization process, ChatGPT not only inserts entities instead of placeholders but also edits the text itself, for example, breaks long sentences, rearranges phrases, and makes other edits. As a result, the added text fragments may contain not only names of the inserted entities but also arbitrary words and phrases. Thus, the proposed approach is not reliable.

As an alternative, we considered another approach: adding an instruction to the pseudonymization prompt to output a list of entities inserted into the text after pseudonymization. However, this approach also proved to be unreliable: the generated list does not always include all inserted entities and may also contain arbitrary text fragments.

Another alternative considered was to add an instruction to the prompt to enclose all inserted entities in curly braces. However, this approach also proved to be unreliable.

As a result, we settled on the following approach. The approach is based on string alignment algorithms used in bioinformatics for aligning nucleotide sequences. A modified version of the Needleman-Wunsch algorithm was used as the alignment algorithm. The proposed approach is as follows: First, align the anonymized and pseudonymized texts. Then, in the pseudonymized text, find fragments that are mapped to the placeholders in this alignment. These fragments are then considered as names of the inserted entities.

In Figure 5 1, a visualization of the proposed approach is shown.

The anonymized and pseudonymized texts are placed opposite each other (with the pseudonymized text below the anonymized text). Non-matching characters are highlighted in red, and gaps are represented by the "-" symbol. Yellow highlights indicate the placeholders in the anonymized text and the corresponding fragments in the pseudonymized text. These fragments are considered as the extracted entities.

```
Оператор: Добрый день, ----# ----#. Клиент: Алло, да. Клиент: Угу. Клиент: У
Оператор: Добрый день, Иван Петров. Клиент: Алло, да. Клиент: Угу. Клиент: У
гу. Клиент: Да. Оператор: Меня зовут ---------- --# -- Сбербанк-, обслуживан
гу. Клиент: Да. Оператор: Меня зовут Елена Сергеева из Сбербанка, обслуживан
ие юридических лиц, звоню по организации ----текста не-т попо------в------
ие юридических лиц. Звоню по организации 'Созвез--здие'. Мыполучиливашу за
-оду--- открытия расчётного счёта- ваш менеджер куратор- --------#----gis пе
явку на открытие расчётного счёта. Ваш менеджер куратор, Андрей Горбунов, пе
редал нам заявку-----------, -о----т----крыв -с------чёт в течение - дней, с
редал нам заявку. Вы сможете получить пак-ет услуг 'GIS' в течение 7 дней. -
можете -получить # gis рекламный пакет— вывеска с возможностью оформления ви
В него вхо--дит- - --- рекламный пакет, вывеска с возможностью оформления ви
трины— скажите, пожалуйста, вам уже рассказывали о наших тарифах- какой-то выб
трины. Скажите, пожалуйста, вам уже рассказывали о наших тарифах? --Вы уже выб
рали пакет? Клиент: Ну, пока начальный. Оператор: -Лёгкий старт-, который бе
рали пакет? Клиент: Ну, пока начальный. Оператор: 'Лёгкий старт', который бе
сплатный, все верно. Клиент: Да. Оператор: Хорошо, подскажите- пожалуйста, в
сплатный, все верно. Клиент: Да. Оператор: Хорошо, подскажите, пожалуйста, в
каком городе планируете открывать счёт? Клиент: В -----#. Оператор: В вашем
каком городе планируете открывать счёт? Клиент: В Москве. Оператор: В вашем
```

Figure 1. Visualization of the entity pseudonymization with LLM. Fictional entities inserted by the LLM are identified and extracted for annotation by aligning the original anonymized transcript with the pseudonymized dialogue.
