# Supplementary material for: SynEL: A synthetic benchmark for entity linking
Source: PLoS One. 2026 Jan 8;21(1):e0339468. doi: 10.1371/journal.pone.0339468 (PMC12782364; doi:10.1371/journal.pone.0339468)
Supplement: S4 Appendix — (PDF) [file pone.0339468.s004.pdf]

S4 Appendix: Named Entity Recognition and Relation Extraction Parameters

List of Extracted Relations

List of Extracted Relations is described in table 1: Relation alignment in the FinRED and REFinD datasets.

Annotating the generated dialogue

System prompt for English-based NER and RE

```
system_message = "You are a helpful assistant, a specialist in relation
extraction and named entity recognition tasks."

inserted_relations = {‘title’, ‘revenue_of’, ‘agreement_with’,
‘telephone_number’, ‘business_division’, ‘chairperson’,
‘chief_executive_officer’, ‘work_industry’, ‘director/manager’,
‘partner_with’, ‘loss_of_money’, ‘profit_of’, ‘employee_of’,
‘employee_num’, ‘cost_of’, ’member_of’, ‘acquired_on’, ‘founded_by’,
‘physical_location’, ‘work_location’, ‘occupation’, ‘legal_form’,
‘owned_by’, ‘owner_of’, ’parent_organization’, ‘position_held’,
‘product_or_material_produced’, ‘subsidiary’}

prompt_ner = f"""Given the dialogue and the corresponding named
entities, your task is to retrieve the relations for these entities
from the given dialogue.
The relations should be retrieved in the json format. The relations
between two entities should be extracted in the format of triplets
listed in json.
The output format should be the following:
[
    [entity_i, entity_j, relation],
    ...
    [],
]
Only the json with the retrieved triplets should be returned.

Each dialogue contains layout of the named entities given there. The
retrieved triplets should contain the entities that are marked in the
dialogue.

First read the dialogue and understand it.
Here is the dialogue for relation extraction from:
{dialogue}"""

prompt_re = f"""Now identify the triplets with relations given the
information below.
Possible relations:
{inserted_relations}
DO NOT include any extra relations (different from listed above) in the
final triplets. Provide outputs in the JSON format. DO NOT include any
explanation for your answer."""
```

**RECustomEN / RE-JoinedEN**	**FinRED**	**REFinD**
no_relation		no_relation
acquired_on		org:date:acquired_on
address		
agreement_with		org:org:agreement_with
business_division	business_division	
chief_executive_officer	chief_executive_officer	
cost_of		org:money:cost_of
director_/_manager	director_/_manager	
employee_num		
employee_of	employer	pers:org:employee_of, pers:univ:employee_of
founded_by	founded_by	pers:org:founder_of
legal_form		
loss_of_money		org:money:loss_of
member_of		pers:org:member_of, pers:gov_agy:member_of, pers:univ:member_of
occupation	distributed_by, manufacturer	
owned_by	owned_by	
owner_of	owner_of	
parent_organization	parent_organization	
partner_with		
physical_location	headquarters_location, location_of_formation	org:gpe:headquartered_in, org:gpe:formed_in
position_held	position_held	
product_or_material_produced	developer, product_or_material_produced	
profit_of		org:money:profit_of
revenue_of		org:money:revenue_of
subsidiary	subsidiary	org:org:subsidiary_of
telephone_number		
title		pers:title:title
work_industry	industry	
work_location		org:gpe:operations_in

Table 1. Relation alignment in the FinRED and REFinD datasets.

System prompt for Russian-based NER and RE

```
user_message = f"""Identify the following named entities in the text
and output them in JSON format:

[(named entity, TYPE), (named entity, TYPE)]

Text: {sample_dialogue}

Entities: {sample_entities}

Provide only JSON with named entities in the end."""

type_prompt = f"""Type is one of the following:
ORG: organization name;
ADDR: address;
NUM: some number (digits);
YEAR: some year;
PERSON: person name;
INDUSTRY: industry of the company work;

Now, provide the JSON answer for the following dialogue:

Text: {dialogue_zero_shot}

Entities: {entities_zero_shot}

Provide only JSON with named entities in the end."""
```

System prompt for Russian-based EL

System prompt for Russian-based EL: All in One

```
user_message = """Про компанию A известны следующие факты:

Вид деятельности: some_industry, Город: some_city, находится по адресу:
some_address, выручка компании: some_revenue, компания связана с
организациями: some_company

Какая из компаний ниже соответствует компании A? Ответ должен содержать
только ИНН

ИНН: some_ID, Вид деятельности: some_industry, находится по адресу:
some_address, выручка компании: some_revenue, компания связана с
организациями: some_company, some_company, some_company

ИНН: some_ID, Вид деятельности: some_industry, находится по адресу:
some_address, выручка компании: some_revenue, компания связана с
организациями: some_company, some_company, some_company

...
```

```
"""
```

System prompt for Russian-based EL: Binary

```
user_message = """
Про компанию A известны следующие факты: Вид деятельности:
some_industry, Город: some_city, находится по адресу: some_address,
выручка компании: some_revenue, компания связана с организациями:
some_company

Вторая компания имеет Вид деятельности: some_industry, находится по
адресу: some_address, выручка компании: some_revenue, компания связана
с организациями: some_company, some_company, some_company

Отвечай да или нет, являются ли эти компании, описанные ниже и той же
организацией?"""
```
